# A new approach to chemotherapy: drug-induced differentiation kills African trypanosomes

**DOI:** 10.1038/srep22451

**Published:** 2016-03-02

**Authors:** Tanja Wenzler, Gabriela Schumann Burkard, Remo S. Schmidt, Pascal Mäser, Andreas Bergner, Isabel Roditi, Reto Brun

**Affiliations:** 1 Medical Parasitology & Infection Biology, Swiss Tropical and Public Health Institute, Basel, Switzerland; 2 University of Basel, Basel, Switzerland; 3 Institute of Cell Biology, University of Bern, Bern, Switzerland; 4 BioFocus DPI, Allschwil, Switzerland

## Abstract

Human African trypanosomiasis (sleeping sickness) is a neglected tropical disease caused by *Trypanosoma brucei* spp. The parasites are transmitted by tsetse flies and adapt to their different hosts and environments by undergoing a series of developmental changes. During differentiation, the trypanosome alters its protein coat. Bloodstream form trypanosomes in humans have a coat of variant surface glycoprotein (VSG) that shields them from the immune system. The procyclic form, the first life-cycle stage to develop in the tsetse fly, replaces the VSG coat by procyclins; these proteins do not protect the parasite from lysis by serum components. Our study exploits the parasite-specific process of differentiation from bloodstream to procyclic forms to screen for potential drug candidates. Using transgenic trypanosomes with a reporter gene in a procyclin locus, we established a whole-cell assay for differentiation in a medium-throughput format. We screened 7,495 drug-like compounds and identified 28 hits that induced expression of the reporter and loss of VSG at concentrations in the low micromolar range. Small molecules that induce differentiation to procyclic forms could facilitate studies on the regulation of differentiation as well as serving as scaffolds for medicinal chemistry for new treatments for sleeping sickness.

African sleeping sickness, also known as human African trypanosomiasis (HAT), is a neglected tropical disease of sub-Saharan Africa caused by two subspecies of protozoan parasites, *Trypanosoma brucei rhodesiense* and *T. b. gambiense.* These are responsible for the acute form of HAT in East Africa and the more chronic form of HAT in Central and West Africa, respectively. *T. b. brucei* causes the disease Nagana in domestic animals, but does not infect humans. All three subspecies of *T. brucei* are transmitted by the bite of an infected tsetse fly, are morphologically indistinguishable and share a very similar life cycle^1^.

African trypanosomes show a number of features that are different from their mammalian hosts including antigenic variation^2^, energy metabolism^3^, polyamine biosynthesis^4^, RNA editing^5^. Differences between a parasite and its host are potentially exploitable as targets for drug discovery. Unfortunately, there is still a large gap between basic and translational research, and the biochemical peculiarities of African trypanosomes have not yet translated into new drug candidates.

The life cycle of *T. brucei* involves the progression through different developmental stages within the insect and the mammalian host. During the differentiation from one life-cycle stage to the next, trypanosomes frequently change their morphology, metabolism and the major surface proteins. There are two types of bloodstream forms in the mammal: the slender form, which is a dividing form, and the stumpy form which is non-dividing and pre-adapted for transmission to the fly. Both bloodstream form stages are covered by a coat of variant surface glycoprotein (VSG), which protects them from both the innate and the adaptive immune system. Only with a full complement of surface VSG are trypanosomes able to survive in the mammalian host. For most of its life-cycle in the tsetse fly vector, the parasite does not require a VSG coat, with the exception of the metacyclic form that is transmitted to a new mammalian host when an infected fly takes a blood meal^6^.

When bloodstream form trypanosomes differentiate to procyclic forms - a process that naturally occurs in the tsetse fly at temperatures lower than 27 °C - they shed their VSG coat and replace it by a procyclin coat^7^. Loss of VSG and gain of procyclin are coupled processes and can also be triggered at 37 °C *in vitro*^8^. Procyclic trypanosomes are highly sensitive to lysis by serum components such as complement factors^9^. A minimally compromised VSG coat suffices to cause rapid clearance of the cells *in vivo*^10^. Thus, untimely differentiation to the procyclic form within the mammalian host would be lethal to the parasite, and a molecule that stimulates this differentiation at 37 °C could be a new type of drug candidate for HAT. Since the mode of action is not based on selective toxicity, the molecule(s) could be expected to have a good safety profile.

The regulation of differentiation is complex, and still not fully understood. Several protein kinases^11^,^12^,^13^,^14^, as well as two protein phosphatases^15^,^16^, are involved in the regulation of differentiation from long slender bloodstream forms to short stumpy or to procyclic forms. These frequently act as molecular brakes; inhibition or down-regulation results in premature progression to the next stage of the life cycle^12^,^13^,^15^,^16^. Differentiation from bloodstream to procyclic forms can be triggered *in vitro* by *cis*-aconitate (CA) or citrate or a combination of the two^17^. The highest efficiency for differentiation is reached starting from short stumpy forms and with a temperature shift from 37 °C to 27 °C. Slender forms also respond to the signal, but differentiation takes longer and is asynchronous. The concentration of citrate/CA required to induce differentiation is temperature-dependent, increasing from 3 mM at 27 °C^17^ to 20 mM at 37 °C^18^. Two other treatments, acid stress^19^ and protease treatment^18^, trigger differentiation of stumpy forms, but not of slender forms.

Our aim is to identify compounds that can induce differentiation of slender trypanosomes at 37 °C at low micromolar concentrations. For this purpose we used transgenic *T. b. brucei* that have the gene for β-glucuronidase (GUS) inserted into a procyclin locus^18^. Expression of the reporter gene *GUS* accurately reflects procyclin expression. GUS activity is absent in bloodstream forms but can be measured in differentiating trypanosomes and in procyclic forms^18^.

In this proof of concept study we first established an *in vitro* assay to assess parasite differentiation at 37 °C in a medium-throughput format, screened close to 7,500 structurally diverse compounds at two concentrations and analysed selected hits for the production of procyclin transcripts and their effects on the VSG coat.

## Results

### Establishment of the GUS differentiation assay and medium-throughput screening

We optimised a whole-cell assay in 96-well format to measure trypanosome differentiation in a medium-throughput format, using a transgenic clone of *T. brucei* (GUSone) that carries the *GUS* gene in one of the procyclin loci^18^. Expression of GUS is used as a proxy for procyclin expression.

**Figure 1:**
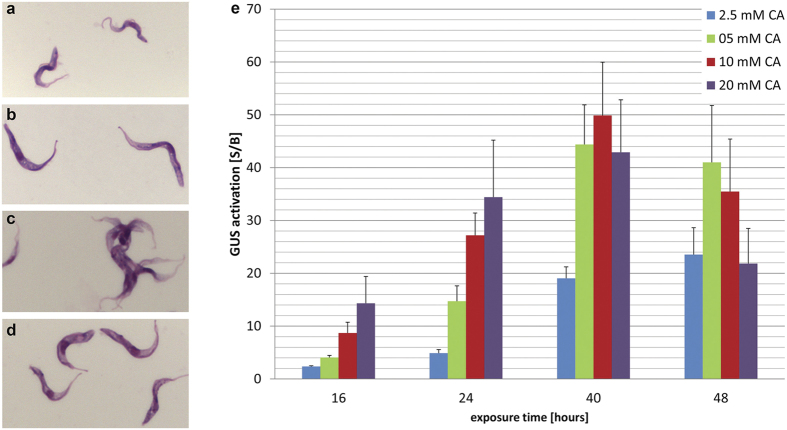
Establishment of GUS assay with CA. Giemsa stained *T. b. b.* GUSone (**a–d**). Bloodstream forms (**a**), procyclic forms (**b**) parasites were differentiated to procyclics with a 24 h exposure to 5 mM CA at 27 °C followed by one week culture in SDM-79 medium at 27 °C. Bloodstream forms that were treated with 10 mM CA for 40 h at 37 °C (**c**), bloodstream forms that were treated with 10 mM CA for 40 h at 27 °C (**d**). GUS activation is dependent on CA concentration and exposure time at 37 °C (**e**). The starting density was 8 × 10^5^ parasites/ml for 16 h and 24 h, 5 × 10^5^ parasites/ml for 40 h, and 2.5 × 10^5^ parasites/ml for 48 h exposure. Bars indicate standard deviations of S/B from at least three independent assays. Cells exposed to CA at 37 °C tend to aggregate. CA = *cis*-aconitate.

Short stumpy forms are non-dividing, have a short lifespan in the mammalian host and differentiate more readily to procyclic forms than long slender trypanosomes. We specifically targeted long slender bloodstream parasites in our screen because they are the relevant forms for chemotherapy. GUSone is a monomorphic trypanosome clone that has lost its ability to develop into short stumpy forms; this guarantees that any differentiation signals obtained are fully attributable to long slender forms. Slender bloodstream forms of GUSone are capable of differentiating to procyclic forms in the presence of 3 mM *cis*-aconitate (CA) combined with a temperature shift to 27 °C^18^. This was confirmed microscopically after Giemsa staining by the cell morphology and the movement of the kinetoplast towards the nucleus (Fig. 1b), as well as by their characteristic motility. At 37 °C, slender bloodstream forms differentiate towards procyclic forms at the level of procyclin or GUS expression^18^. However, Giemsa staining revealed that, morphologically, the parasites were not genuine procyclic forms (Fig. 1c). Moreover, agglutination of trypanosomes was observed in the presence of heat-inactivated horse serum, which might be indicative of surface coat changes (see below). The GUS signal to background ratio (S/B) was also lower at 37 °C than at 27 °C.

Cultured bloodstream forms and parasites harvested from mice were compared in the assay. The S/B ratio was higher with cultured bloodstream forms at comparable CA concentrations (34 versus 19 after 24 h exposure). As a consequence, assay conditions and parameters were optimised at 37 °C with CA and cultured bloodstream forms. GUS activity was dose- and time-dependent (Fig. 1e). The signal increased for up to 40 h exposure to CA at concentrations ranging from 2.5 to 20 mM. At longer exposure times, and at concentrations >5 mM CA, the GUS differentiation signal decreased on account of cell death. The IC_50_ values were ≥20 mM up to 40 h exposure to CA, and 8 mM for 48 h exposure time in the Alamar blue viability assay^20^, using the same initial trypanosome density as in the GUS assay.

The final parameters chosen for the medium-throughput screen were 7 × 10^5^ GUSone bloodstream forms/ml, and 40 hours drug exposure time followed by 2–3 hours incubation with the GUS substrate 4-methylumbelliferyl-beta-D-glucuronide (MUG), all at 37 °C. 10 mM CA was used as a positive control and trypanosomes without any test compound as the background control. The concentration of DMSO (in which all test compounds were dissolved) was kept at 1% throughout the assays. The plates were read before the addition of the substrate, in order to record the innate fluorescence of each test compound and 3 hours after addition of MUG. This procedure allowed us to avoid false positive results.

Four iterative rounds of screening took place. An overview of the four batches is given in Table 1. The assay was highly robust with a mean Z’-factor of 0.91 (Fig. S1). In total, 7,495 compounds were tested. Batch 1 was screened at concentrations of 2 and 20 μg/ml; this was decreased for subsequent batches to 1 and 10 μg/ml. The majority of test compounds were selected based on chemical diversity. However, knowledge about potentially active structures, properties and signalling pathways was also incorporated into the selection process. Batch 1 comprised 1,975 diverse compounds and 25 analogues of *cis*-aconitate and citrate. A total of 35 hits were found in batch 1, corresponding to an overall hit-rate of 1.8%. No active analogues of CA or citrate were identified, in all likelihood because the test compounds were screened at concentrations 10^3^–10^4^-fold lower than the concentration of CA that is required to trigger differentiation at 37 °C. Batch 2 comprised 77 iron chelator-like compounds (since citrate and CA are metal chelators) and an additional 1,923 diverse drug-like compounds. Screening of batch 2 resulted in 30 hits, corresponding to an overall hit-rate of 1.5%, very close to the hit-rate found in batch 1. Only two of the iron chelators were found to be active, corresponding to 6.7% (the higher hit-rate is statistically not significant, given the small hit number).

Activity data from batches 1 and 2 resulted in the identification of 101 structures active in the GUS assay. These were utilised for the selection of batch 3 using computational chemistry hit expansion methods^21^. Three subsets were derived using different computational chemistry methods: Bayesian learning (1,250 compounds), fingerprint-based (500 compounds) and feature-tree based (1,250 compounds) similarity searches. Screening of batch 3 resulted in 159 hits corresponding to a higher hit-rate of 5.3% compared to the two previous batches.

Batch 4 contained 495 compounds that were similar to known inhibitors of MAP kinases. The rationale behind this was that trypanosomes lacking TbMAPK5 show enhanced differentiation to stumpy forms^12^. Nineteen hits were obtained from screening this set, resulting in a hit-rate of 3.8%.

### Hits for drug-induced differentiation cause cell death

**Figure 2:**
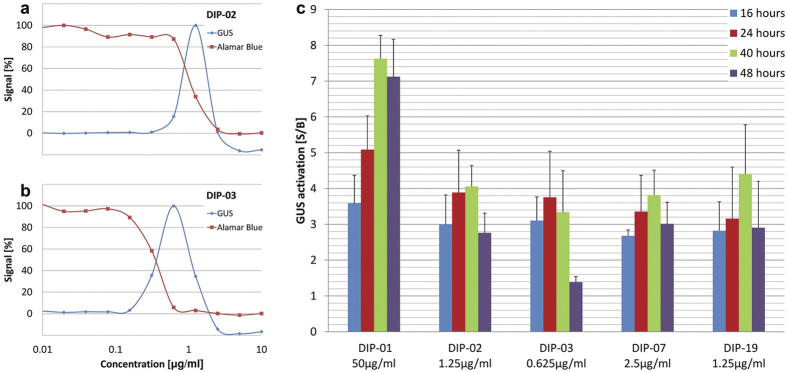
GUS differentiation signal is time- and concentration-dependent and coincides with cell death. Serial dilutions of DIP-02 (**a**) and DIP-03 (**b**) in the GUS differentiation assay and the Alamar blue viability assay (40 h exposure time). GUS activation (S/B) of DIP-01, DIP-02, DIP-03, DIP-07 and DIP-19 at different exposure times (16 h, 24 h, 40 h and 48 h) at the concentration for optimal GUS expression (**c**). Bars indicate standard deviations of S/B from at least three independent assays. CA = *cis*-aconitate.

In total, 243 hits induced GUS expression >2 fold, including 45 at the lower concentration. None produced as high a signal as 10 mM CA. However, all compounds induced GUS expression at concentrations that were up to 5,000-fold lower than CA. Of the hits, 212 were titrated to measure their concentration-dependence in two assays (Table S1). One was the GUS assay itself, to determine the concentration yielding the highest activation signal. The second assay was the Alamar blue viability assay to determine the IC_50_ of each compound. Representative curves for two compounds are shown in Fig. 2. GUS activity was both concentration-dependent (Fig. 2a,b) and time-dependent (Fig. 2c). Bayesian active feature learning revealed several substructure features that were significantly enriched in the 212 active compounds, compared to the total set of 7,495 (Fig. S2).

Our original hypothesis was that compounds that trigger differentiation would probably not be trypanocidal *in vitro*. The dose-response curves of all the GUS assays were bell-shaped, however, and not sigmoidal (Fig. 2), indicating that higher concentrations of the compounds were inhibitory. These findings were verified by the Alamar blue assays, which revealed that all 212 hits showed trypanocidal activity that overlapped to a greater or lesser extent with activation of GUS expression (Fig. 2 and Table S1). Superimposing these two properties results in the bell-shaped curves observed. Although the compounds that induced GUS expression also killed trypanosomes, the reverse was not true. Of 2,231 compounds that caused ≥70% growth inhibition at the higher concentration of 20 μg/ml or 10 μg/ml in the Alamar blue viability assay, only 9% showed GUS activation at either of the two concentrations tested. Thus 91% of the compounds that were trypanocidal/trypanostatic did not activate expression of the reporter gene. Among a panel of reference drugs for HAT, only melarsoprol produced a GUS signal (Table 2, Figs S3 and S4).

The dose response results were used to select 30 compounds that showed a strong GUS signal, preferably at a low concentration and with a reduced overlap with trypanocidal activity at the concentration resulting in the highest GUS activity. After purity analyses, 28 compounds were retained for further experiments (Table 2). The structures are shown in Fig. S5. All compounds were from the Biofocus open access library and could be purchased and used without any restrictions. These were designated DIP-01 to DIP-28 (DIP for Drug Induced differentiation towards Procyclics).

The 28 DIP compounds were also tested in the Alamar blue viability assay using a non-transgenic *T. b. rhodesiense* reference strain, STIB900, and *T. b. gambiense* strain STIB930. None of them exhibited higher IC_50_ values than against the transgenic GUSone clone with exception of DIP-13 on *T. b. gambiense* (Table S2). This indicates that the trypanocidal activity is inherent to the DIP hits and independent of GUS activity. Moreover, trypanocidal activity did not require complement or other heat-labile factors as it also occurred with heat-inactivated foetal calf serum. In addition, killing was not enhanced by the addition of guinea pig complement (data not shown).

### Compounds inducing differentiation kill rapidly

For further analysis we selected 4 compounds (DIP-02, -03, -07 and -19) that induced GUS activity with a S/B ratio >3 at concentrations of ≤2.5 μg/ml. GUS-01 was also selected as it gave the highest S/B ratio of all DIP compounds tested (7.6), and it showed less overlap with cell death.

According to the Alamar blue viability assay the DIP hits were fast acting. IC_50_ values of the 5 selected compounds did not change with increasing exposure times from 16 to 48 hours (Table 3). Among the existing drugs for the treatment of African sleeping sickness, melarsoprol is the only one that acts as fast^22^. Interestingly, melarsoprol shows GUS activation as well (S/B = 2 see Fig. S4). An independent confirmation of the short time to kill was provided by real-time isothermal microcalorimetry (Figs S6 and S7). Finally, the minimal concentration required to kill trypanosomes within 24 hours was determined microscopically (Table 3). The difference between the IC_50_ and MIC was significantly smaller for DIP compounds and melarsoprol than for pentamidine. These experiments confirmed that DIP-02, -03, -07, -19, like melarsoprol, rapidly induce cell death.

### Loss of VSG and procyclin expression induced by selected DIP compounds

**Figure 3:**
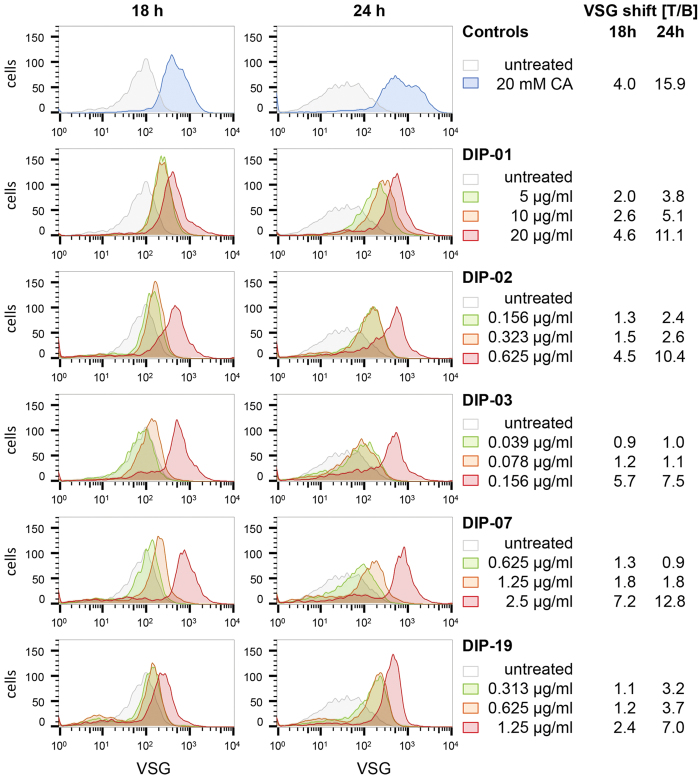
Loss of VSG after treatment with 20 mM CA or various concentrations of DIP compounds at 37 °C. The density of the VSG coat was monitored by staining with anti-VSG221 antibodies and flow cytometry. All assays were performed in triplicate, using one 96-well plate for each time-point (18 and 24 h); the untreated and CA-treated samples on each plate thus serve as controls for the whole series measured per time-point. To display the dose-dependent VSG shifts, representative curves of different DIP concentrations were plotted together with the corresponding untreated control. MFI: median fluorescence intensity. T/B: VSG changes are expressed as a ratio of the MFI after treatment to the MFI of the untreated control. Full flow cytometry data are available in Table S4.

The GUS assay identifies compounds that induce procyclin expression, but provides no information about the VSG coat, which is normally shed when trypanosomes differentiate. To monitor the status of the coat after exposure to various compounds, trypanosomes were labelled with polyclonal anti-VSG antibodies and analysed by flow cytometry. The VSG molecules on the surface of bloodstream forms are so tightly packed that only a limited number of epitopes are accessible for binding. As some VSG is shed, the coat loosens and more epitopes are exposed, resulting in more antibody binding^23^ and an increase in the median fluorescence intensity (MFI). Binding to untreated bloodstream forms is defined as background binding (B). Changes are expressed as a ratio of binding after treatment to binding without treatment (T/B). The five DIP compounds were tested at different concentrations and two exposure times (Fig. 3). Trypanosomes treated with CA (positive control) gave T/B ratios of 4.0 (at 18 h) and 15.9 (at 24 h) compared to untreated cells (Fig. 3, Table S3). Trypanosomes treated with the selected DIP compounds, showed dose-dependent loss of VSG comparable to treatment with CA. The DIP compounds were considerably more potent however. For example, 0.5 μM (0.156 μg/ml) DIP-03 and 10 and 20 mM CA showed similar shifts in fluorescence intensity (Fig. 3 and Table S3 and S4).

We also monitored VSG in cells treated with melarsoprol, which showed low level GUS activity at high drug concentrations, and pentamidine and nifurtimox, which were GUS-negative at all concentrations tested (Table 2, Table S5 and Fig. S8). All three compounds exhibited VSG loss at concentrations ≥IC_50_. Thus VSG loss is not restricted to differentiating cells, but also occurs in dying cells.

**Figure 4:**
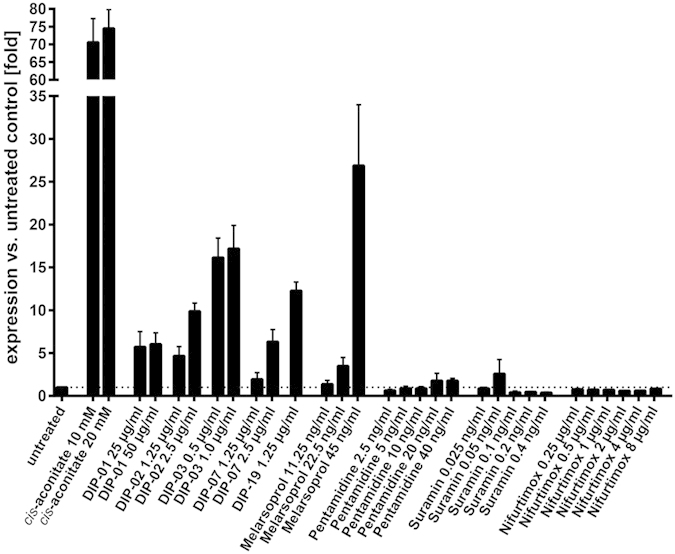
Real-time qPCR to measure procyclin expression levels. GUSone bloodstream forms were exposed for 24 hours to the 5 DIP compounds DIP-01, DIP-02, DIP-03, DIP-07 and DIP-19 and to CA at 37 °C. Procyclin mRNA levels were determined by qPCR. Among the standard trypanocidal drugs only melarsoprol induced expression of procyclin mRNA above background level. Error bars show standard deviations for technical triplicates.

To investigate whether differentiation might be a stress response to drug treatment, we measured procyclin transcripts by quantitative real time PCR. This method is highly sensitive, but not feasible for large-scale screens. Figure 4 shows that CA and all 5 DIP compounds trigger procyclin expression. With the exception of melarsoprol, which also induced GUS, the trypanocidal drugs did not induce procyclin expression over a range of concentrations above and below the IC_50_. These results confirm the specificity of the DIP compounds as triggers of differentiation.

## Discussion

Here we present a new chemotherapeutic strategy against African trypanosomes: premature differentiation at 37 °C of the mammalian bloodstream forms into the procyclic forms that normally first develop in the tsetse fly vector. The direct differentiation of proliferating bloodstream form to procyclic trypanosomes is an unphysiological process. In nature, only the cell cycle-arrested stumpy forms are competent to undergo this differentiation, which takes place over 2–3 days at ≤27 °C in the midgut of the tsetse fly^24^. Thus our strategy is fundamentally different from other approaches. In theory, triggering the loss of VSG, with a concomitant expression of procyclin would make the parasites vulnerable to the human innate immune system.

**Figure 5:**
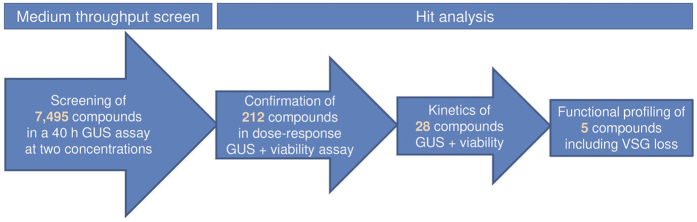
Overview of the screening procedure to identify compounds triggering differentiation. 7,495 compounds were screened at two concentrations in the GUS assay. 243 compounds induced GUS with a S/B >2-fold, of which 212 available compounds were subjected to dose-response analyses in the GUS and viability assays. 30 compounds induced GUS activity S/B >3-fold of which 28 compounds were available and highly pure. Five compounds were further analysed by flow cytometry (loss of VSG) and real-time quantitative PCR (induction of procyclin mRNA).

Using a reporter gene as a proxy for procyclin expression, we established an *in vitro* screen for small molecules that induce differentiation based on a *T. brucei* reporter line that expresses β-glucuronidase (GUS) under the control of a procyclin promoter^18^. This whole-cell assay was highly robust with a Z’-factor of 0.9 (Fig. S1) and amenable to screening in 96-well plates. We performed a medium-throughput screen with 7,495 compounds that were tested at 2 concentrations. 212 of the hits were titrated in the GUS differentiation assay and in the Alamar blue viability assay and based on the dose-response curves, 28 DIP hits were selected for further characterisation (Fig. 5). *Cis*-aconitate served as a positive control that strongly induces GUS expression, albeit at millimolar concentrations^18^ (Fig. 1e). Comprehensive characterisation of 5 selected hits showed that all of them induced expression of GUS protein and procyclin mRNA (Fig. 4). This did not occur with pentamidine, suramin or nifurtimox, indicating that it is a specific part of the differentiation program and not a stress response of dying cells. In contrast to the induction of GUS and procyclin expression, we found that loosening of the VSG coat is a more general phenomenon when cells are exposed to high concentrations of trypanocidal drugs. Although cells actively shed VSG as part of the differentiation program^7^, it can also be released through the action of GPI-phospholipase C when cells disintegrate^25^. Since all the DIP hits were trypanocidal *in vitro*, as demonstrated by Alamar blue assays (Table 2), it is difficult to disentangle the two processes.

Killing by DIP hits occurred fast, as monitored in real time by isothermal microcalorimetry (Fig. S6). Trypanosomes were highly sensitive to these compounds when cultured with heat-inactivated serum, negating our initial hypothesis that serum components such as complement are required for lysis. Thus the forced differentiation of proliferating bloodstream forms to procyclic-like forms at 37 °C seems to lead to a form of programmed cell death that is independent of heat-labile factors. This is in agreement with the recent finding that the down-regulation of repressor of differentiation kinase 2 (RDK2) in bloodstream forms of *T. brucei* resulted in procyclin expression and cell death^13^. The trypanocidal activity of the DIP hits resulted in bell-shaped dose-response curves for GUS expression (Fig. 2), because at higher concentrations these compounds killed the trypanosomes too fast to allow for substantial GUS production. For this reason we cannot exclude that more potent triggers of differentiation were present, but GUS activity would not have been detected at the concentrations tested in the medium-throughput screen. Though all GUS inducing compounds were trypanocidal, the majority of the trypanocidal compounds did not induce GUS expression. Thus, activation of differentiation to procyclic forms is not a default pathway in dying cells.

Drug resistance is a constant threat to the efficacy of antiparasitic drugs. Cross resistance between current drugs has been reported by shared uptake routes (melarsoprol and diamidines via P2 transporter or AQP2)^26^ and cross resistance between fexinidazole and nifurtimox could emerge based on their shared requirement for drug activation by nitroreductases^27^. With the screening strategy described here, we are likely to identify different chemotypes, and with the new mechanism of action, hits will bear a low risk of showing cross-resistance to existing antitrypanosomal drugs. Another advantage of the compounds triggering differentiation is their rapid trypanocidal activity.

In summary, we present a new approach to combat parasites that progress through different life-cycle stages and have validated it with a medium-throughput screen on *T. brucei* for small molecules that could induce abortive differentiation of bloodstream to procyclic forms in the mammalian host. In principle, this concept could also be applied to other parasites with complex life cycles.

## Methods

### Materials

*Cis*-aconitate (CA, SIGMA) was dissolved in water and the pH was adjusted with NaOH to pH 7.4. MUG solution: 352 mg/l 4-Methyl-Umbelliferyl-beta-D Glucuronide (Promega/Serva) was dissolved in lysis buffer consisting of 328 ml/l 1 M Tris-HCl (pH 8), 120 mg BSA, 24 ml/l 10% SDS, 648 ml/l H_2_O. Resazurin (SIGMA) was dissolved in water (12.5 mg/100 ml). 96-well microtitre plates were from Corning.

### Compound selection

All compounds were selected from the BioFocus diverse compounds collection (BFDCC), or the BioFocus lead-like collection (BFLLC). At the time of the project, the BFDCC and BFLLC comprised approximately 850,000 and 180,000 compounds, respectively. The majority of the 7,495 tested compounds were selected based on chemical diversity. Additional compounds were analogues of *cis*-aconitate and citrate, iron chelator-like and MAP kinase (ERK) inhibitor-like compounds. The screening and compound selection was carried out in four batches (Table 1). Batch 4 consisted of 495 compounds that were similar to known ERK inhibitors in the ChemBL database^28^.

Compounds were supplied in 96-well plates in 10 μl MEM containing 10% DMSO (resulting in 1% final concentration) for the medium throughput screening for GUS activation. The 212 selected compounds were provided from Biofocus in two sets for dose response analysis in the GUS differentiation assay and the Alamar Blue viability assay. The 28 hits (DIP compounds) can be found via the Pubchem ID (Table 2) (http://pubchem.ncbi.nlm.nih.gov).

### *Trypanosoma brucei* cell line and cultivation

The transgenic *T. b. brucei* clone GUSone has been described previously^18^. The coding region of one procyclin gene was replaced by *E. coli* β-glucuronidase (GUS). The GUS gene reacts in parallel to procyclin genes, which are expressed when the trypanosomes start to differentiate^18^. The parasites were cultured and screened in MEM supplemented according to Baltz *et al.*^29^ with minor modifications: 0.2 mM 2-mercaptoethanol, 1 mM sodium pyruvate, 0.5 mM hypoxanthine, and 15% heat-inactivated horse serum and incubated at 37 °C in a humidified atmosphere containing 5% CO_2_. The identical medium was used for *T. b. rhodesiense* strain STIB900, a derivative of strain STIB704^30^. The medium was modified for the *T. b. gambiense* strain STIB930^31^ by replacing the horse serum with inactivated 5% human serum and 15% FBS. For the loss of VSG assay, New York single-marker (NYsm) bloodstream forms^32^ expressing VSG221 were cultivated in HMI-9 medium containing 10% FBS at 37 °C in a humidified atmosphere containing 5% CO_2_^33^.

### GUS differentiation assay

Compounds were provided by BioFocus in Costar^TM^ 96-well microtitre plates. Tests were run in parallel with concentrations of 20 μg/ml and 2 μg/ml (batch 1) or 10 μg/ml and 1 μg/ml (batches 2–4). The medium of GUSone culture with a density of approximately 10^6 ^parasites/ml was replaced with fresh culture medium. For this the cells were collected by centrifugation for 10 minutes at 3,000 rpm (1840 g) at 37 °C and the cells resuspended in fresh culture medium (warmed up to 37 °C) to the desired parasite density. In differentiation studies considerably higher cell densities are required compared to growth inhibition assays. The parasite suspension was added to each well, resulting in 7 × 10^4 ^parasites/100 μl, and the plates incubated for 40 hours at 37 °C in a humidified atmosphere containing 5% CO_2_. After 40 hours drug exposure time, the plates were read to assess intrinsic fluorescence of the compounds at an excitation wavelength of 362 nm and an emission wavelength of 450 nm with the fluorescence reader Spectramax Gemini XS microplate fluorometer (Molecular Devices Corporation, Sunnyvale, CA). 100 μl MUG solution was added to each well and 3 hours after substrate addition, the plates were read again at identical wavelengths to measure GUS activity. The first reading was used to detect autofluorescence of compounds that would cause false positive results. Each plate contained control wells: 10 mM *cis*-aconitate served as a positive control (differentiation control) and wells with trypanosomes without any drug served as a negative control (background).

### Alamar blue assay

The 50% inhibitory concentrations (IC_50_) were determined as previously described^22^ with minor adaptations. The Alamar blue assay was set up in parallel to the GUS assay, using the same medium and compound concentrations. The parasite density was adjusted to the different incubation times all in fresh culture medium. The starting density was 8 × 10^5^ parasites/ml for 16 h and 24 h, 5 × 10^5^ parasites/ml for 40 h and 2.5 × 10^5^ parasites/ml for 48 h drug exposure. Trypanosomes without any drug served as positive controls (100% parasite growth) and culture medium without parasites served as a negative control. The plates were read with a Spectramax Gemini XS 4 hours after addition of resazurin with an excitation wavelength of 536 nm and an emission wavelength of 588 nm.

### Microcalorimetry to monitor time of drug action

Time of drug action was monitored in real-time using isothermal microcalorimetry^34^. The heat flow data can be used as a proxy for the number of viable cells. Onset of drug action can be determined by a reduced metabolic activity of drug treated cells compared to untreated control. Time to kill of the parasite population is reached when the heat flow is down to baseline. Compared to the previous studies^22^,^35^ we reduced the volume of the samples to avoid the oscillations that appear at high starting densities of trypanosomes^34^ and optimised conditions for fast acting compounds (0.5 ml containing 4 × 10^5^ GUSone bloodstream forms). With the high trypanosome density we could monitor not only growth inhibition or growth arrest but also trypanocidal activity. The cell suspensions were spiked with different concentrations of DIP compounds, with a final DMSO concentration of 0.1 or 0.3% (vl/vl) depending on the concentration tested. Each sample was set up in duplicate. The heat flow was continuously monitored at 37 °C in the isothermal microcalorimeter (thermal activity monitor, model 249 TAM III; TA instruments, New Castle, DE).

### Loss of VSG assay and flow cytometric analysis

Triplicate samples of 300 μl medium with NYsm cells^32^ at a density of 2 × 10^5^/ml and different concentrations of compounds were transferred to a standard 96-well plate and incubated for 18 h or 24 h, respectively, at 37 °C. For flow cytometric analysis, live cells were stained according to the following protocol: cultures were transferred into a V-bottom 96-well plate and the cells were collected by centrifugation for 8 minutes at 1,300 g at 4 °C. The cells were washed once with 150 μl ice-cold HMI-9 (without serum) and then incubated for 30 minutes on ice in 50 μl of rabbit anti-VSG221 polyclonal antiserum (a gift from Jay Bangs, University of Buffalo, USA) diluted 1:20,000 in cold HMI-9. Cooling of the samples avoided internalisation occurring at 37 °C^36^. After washing twice with 150 μl cold HMI-9, the secondary antibody goat anti-rabbit Alexa Fluor 488 (Invitrogen; diluted 1:1000) was added in 50 μl cold HMI-9 medium. Cells were washed twice with 150 μl cold PBS, resuspended in 150 μl PBS and transferred to U-bottom 96-well plates. After addition of another 50 μl of PBS fluorescence was measured using a BD FACSArray^TM^ Bioanalyzer and analysed with the software FlowJo v10.0.8. The acquired data of 5000 counted cells per sample was plotted in histograms (without gate) and the average of the median fluorescence intensities (MFI) was calculated per triplicate sample. The MFI of the drug treated samples (T) was compared with the MFI of untreated samples (background, B).

### Messenger RNA quantification

GUSone cells were exposed to different concentrations of compounds for 24 hours as described above. Cells were collected by centrifugation, washed in PBS and pelleted. After removal of the supernatant the cells were shock frozen in liquid nitrogen and stored at −80 °C. After thawing the cell pellets, RNA was isolated using an RNeasy Mini Kit (Qiagen, Hilden, Germany) in combination with on-column RNase-free DNase (Qiagen) treatment, following the manufacturer’s protocols. Subsequently, 500 ng total RNA was used for generation of complementary DNA using SuperScript Reverse Transcriptase (Invitrogen). Subsequently, qPCR reactions were prepared using the Power SYBR Green PCR master mix system (Applied Biosystems). Primers were used at 150 nM final concentration; cDNA template was added at 0.5 μl per 12 μl reaction. Assays were carried out in an Applied Biosystems StepOnePlus device. C1 was used as a control gene for normalisation^13^.The primer pairs used in this study are summarized in Table S6. All assays were carried out at least twice. Within an experiment, technical triplicates were run in parallel.

## Additional Information

**How to cite this article**: Wenzler, T. *et al.* A new approach to chemotherapy: drug-induced differentiation kills African trypanosomes. *Sci. Rep.* **6**, 22451; doi: 10.1038/srep22451 (2016).

## Supplementary Material

Supplementary Information

## Figures and Tables

**Table 1:** Overview of screening batches with hit rates.

Batch	Sizetotal	Bias	Sizebias	Hits	Hits bias	Hit-rateper batch	Hit-ratebias
1	2,000	*cis*-aconitate	25	35	0	1.8	0.0
2	2,000	iron chelators	77	30	2	1.5	6.7
3	3,000	hit expansion	3,000	159	159	5.3	5.3
4	495	ERK inhibitors	495	19	19	3.8	3.8
total	7,495			243		3.2	

Compounds were sourced from Biofocus DPI.

**Table 2:** Selected hits.

Pubchem name	Compound	GUS activity[S/B]	optimal conc.for GUS [μg/ml]	Viability IC_50_[μg/ml]
	*cis*-aconitate	58.3	10	>20
STOCK4S-04391	DIP-01	7.6	50	57.2
STOCK1S-31919	DIP-02	4.2	1.25	1.0
STOCK1S-42137	DIP-03	3.3	0.5	0.3
STOCK1S-13735	DIP-04	8.1	50	25.8
STOCK4S-90544	DIP-05	3.3	12.5	7.7
STOCK3S-11937	DIP-06	4.6	6.3	1.3
STOCK5S-42531	DIP-07	3.8	2.5	2.2
STOCK5S-38263	DIP-08	3.5	12.5	5.5
STOCK5S-44766	DIP-09	3.2	50	16.4
STOCK5S-82907	DIP-10	4.4	3.0	1.7
STOCK5S-91387	DIP-11	3.6	12.5	5.8
STOCK5S-83458	DIP-12	3.1	12.5	10.0
STOCK6S-01740	DIP-13	5.4	75	13.4
STOCK6S-10803	DIP-14	3.3	50	22.5
STOCK6S-14165	DIP-15	5.1	25	9.5
STOCK6S-12339	DIP-16	3.4	12.5	8.8
STOCK6S-10284	DIP-17	3.0	28.1	13.9
STOCK6S-06338	DIP-18	3.6	12.5	6.3
STOCK6S-38758	DIP-19	4.4	1.25	0.8
STOCK6S-34290	DIP-20	3.3	10.9	8.6
STOCK6S-35180	DIP-21	3.4	6.3	4.2
5740240	DIP-22	5.0	9.4	4.6
5742784	DIP-23	3.7	21.9	13.9
T5700438	DIP-24	3.5	3.9	2.3
T6214849	DIP-25	3.4	10.9	6.7
T5314847	DIP-26	5.1	10.9	5.3
T5845297	DIP-27	3.0	9.7	4.6
T5227647	DIP-28	3.9	20	10.9
	Melarsoprol	2.1	0.023	0.016
	Pentamidine	1.0	NA	0.006
	Suramin	1.0	NA	0.08
	Nifurtimox	1.0	NA	1.0
	Fexinidazole	1.0	NA	2.4

GUS activity S/B = ratio of GUS signal of drug-treated samples to untreated (background), at the optimal concentration for GUS activity, IC_50_: inhibitory concentration resulting in 50% parasite death. NA: not applicable (there was no GUS signal). Each control compound was tested over a ≥1,000 fold concentration range in both assays with the IC_50_ approximately in the middle of the range. All assays were performed with an exposure time of 40 h; the initial parasite density was 5 × 10^5^/ml in both assays. STOCK5S-42531 is desloratidine.

**Table 3:** IC_50_s in the Alamar blue viability assay using the *T. b. brucei* clone GUSone with different exposure times from 16 h to 48 h.

Compound	IC_50_[μg/ml]				MIC [μg/ml]
	16 h	24 h	40 h	48 h	24 h
DIP-01	75.6	70.1	57.2	47.1	>100
DIP-02	1.2	1.1	1.1	1.2	10
DIP-03	0.4	0.4	0.4	0.4	2.5
DIP-07	2.2	2.0	2.2	1.9	20
DIP-19	0.9	0.9	0.8	0.8	20
Melarsoprol	0.021	0.020	0.016	0.012	0.25
Pentamidine	0.018	0.012	0.006	0.003	>20
CA	>20 mM	>20 mM	20 mM	8 mM	>20 mM

Minimum Concentration to kill all trypanosomes (MIC) was determined microscopically after 24 h of exposure. Assays were performed starting with 4 × 10^5^ trypanosomes in 0.5 ml. CA: *cis*-aconitate 20 mM = 3.48 mg/ml.
