# Supplementary material for: A new approach to chemotherapy: drug-induced differentiation kills African trypanosomes
Source: Sci Rep. 2016 Mar 2;6:22451. doi: 10.1038/srep22451 (PMC4773815; doi:10.1038/srep22451)
Supplement: Supplementary Information [file srep22451-s1.pdf]

A new approach to chemotherapy: drug-induced differentiation kills African trypanosomes

Tanja Wenzler (1,2), Gabriela Schumann Burkard (3), Remo Schmidt (1,2), Pascal Mäser (1,2), Andreas Bergner (4), Isabel Roditi (3), Reto Brun (1,2).

(1) Medical Parasitology & Infection Biology, Swiss Tropical and Public Health Institute, Basel, Switzerland
(2) University of Basel, Basel, Switzerland
(3) Institute of Cell Biology, University of Bern, Bern, Switzerland
(4) BioFocus DPI, Allschwil, Switzerland (present address: Boehringer Ingelheim RCV, Vienna, Austria)

Supplementary information tables

Table S1. 212 hits and the data from the GUS differentiation and Alamar blue viability assays. GUS activity S/B = GUS signal of drug treated samples to signal of background samples, optimal concentration for GUS activation, IC_{50}: 50% inhibitory concentration. NA: not applicable (there was no GUS signal). All assays were performed with a 40 h exposure time on *T. b. brucei.* GUSone. The initial parasite density was $7×10^5$/ml in the GUS assay and $2.5×10^5$/ml in the Alamar blue assay.

PubChem ID	GUS activity [S/B]	optimal conc. for GUS [µg/ml]	Viability IC_{50} [µg/ml]
405_5923_0447	1.7	25	34.4
582_5690_8152	2.9	12.5	14.2
628_8463_9573	5.3	12.5	18.8
5105854	1.3	25	27.2
5259437	3.2	25	22.6
5352515	4.6	6.25	8.0
5426064	1.7	12.5	25.0
5527585	2.5	25	>50
5729045	2.9	12.5	19.5
5569698	3.8	12.5	18.4
5734991	3.8	25	21.0
5740240	5.8	6.25	8.6
5742422	4.6	25	23.6
5742784	3.8	12.5	18.1
6367347	1.9	25	25.8
5879554	3.1	25	32.8
7493764	2.5	6.25	15.7
95703437	2.9	12.5	13.0
BTB 14532	4.0	25	39.6
CD 05692	1.2	12.5	26.0
8L-729	1.2	12.5	27.8
10L-630S	1.1	NA	>50
2K-091	1.7	12.5	22.0
STOCK3S-02777	3.4	25	28.9
STOCK2S-80114	1.8	25	25.3
STOCK2S-82575	2.9	6.25	13.3
STOCK2S-88445	1.9	25	12.8
STOCK2S-89152	1.8	25	26.9
STOCK2S-96769	1.0	NA	31.5
STOCK2S-08783	2.6	6.25	12.6
STOCK1S-31097	2.7	12.5	20.9

STOCK1S-83980	3.4	12.5	20.8
STOCK2S-40414	3.0	25	18.3
STOCK2S-02216	1.3	6.25	7.5
STOCK1S-06041	2.4	25	24.4
STOCK3S-27791	2.8	12.5	23.5
STOCK3S-28703	2.2	25	32.6
STOCK1S-09923	3.8	3.125	1.9
STOCK2S-13266	1.5	12.5	18.9
STOCK1S-23169	1.2	12.5	18.9
STOCK3S-17959	1.9	6.25	24.5
STOCK3S-36150	4.4	12.5	10.4
STOCK3S-44534	4.5	12.5	10.9
STOCK3S-81258	2.2	6.25	13.6
STOCK4S-16503	1.7	12.5	11.4
STOCK1S-93356	1.6	25	28.4
STOCK2S-38696	1.2	3.125	4.6
STOCK2S-14011	2.2	6.25	10.1
STOCK5S-44811	2.6	12.5	25.6
STOCK5S-48033	2.0	25	23.7
STOCK5S-48081	1.5	25	>50
STOCK5S-44766	3.0	12.5	25.6
STOCK5S-54405	2.9	25	20.0
STOCK5S-55771	3.4	12.5	10.4
STOCK5S-77305	1.4	12.5	18.0
STOCK5S-76783	2.2	6.25	16.3
STOCK5S-72614	1.4	6.25	12.9
STOCK5S-91387	5.2	12.5	10.3
STOCK5S-68323	4.6	12.5	12.3
STOCK5S-80549	3.2	12.5	14.3
STOCK5S-83458	4.2	12.5	11.6
STOCK5S-89031	2.4	12.5	19.2
STOCK5S-96369	2.1	6.25	9.8
STOCK6S-01740	6.1	25	32.6
STOCK5S-50422	2.5	12.5	16.9
STOCK5S-57687	1.7	25	25.8
STOCK5S-89689	3.6	6.25	6.1
STOCK5S-92524	2.3	12.5	18.2
STOCK5S-90569	2.7	12.5	12.4
STOCK5S-79972	1.6	25	26.4
STOCK6S-14101	1.3	25	>50
STOCK6S-12050	1.4	25	>50
STOCK6S-10803	4.8	12.5	23.4
STOCK6S-14165	5.6	6.25	14.6
STOCK6S-12339	3.4	6.25	15.6
STOCK6S-10284	4.1	6.25	17.1
STOCK6S-06338	3.5	6.25	15.3
STOCK6S-09080	2.2	12.5	25.4
STOCK6S-18317	3.0	12.5	14.3
STOCK6S-22702	2.2	25	48.8
STOCK6S-27511	3.5	25	41.0
STOCK6S-40884	0.9	NA	50.9
STOCK6S-34290	3.1	6.25	7.7
STOCK1N-21603	2.3	6.25	7.3
AE-641/30156002	3.9	6.25	7.1
AH-034/08306008	1.2	25	45.2
AG-690/36516036	1.0	NA	>50
AG-690/36644020	1.0	NA	>50
AK-249/36810004	1.2	25	>50

AC-907/34131044	0.9	NA	>50
AE-842/34026055	1.2	25	30.4
AE-842/34646010	0.9	NA	40.8
STOCK4S-90544	3.4	3.125	8.1
STOCK4S-57232	3.7	12.5	13.6
STOCK4S-54253	4.1	12.5	10.8
STOCK4S-82648	1.3	25	26.1
STOCK5S-20823	1.7	25	19.6
STOCK5S-06163	2.9	6.25	7.3
STOCK3S-37136	2.9	6.25	7.0
STOCK5S-49612	1.4	3.125	4.4
STOCK4S-66388	0.9	NA	26.4
STOCK5S-13066	2.8	25	33.1
STOCK5S-18906	2.2	12.5	28.9
STOCK5S-25534	1.4	12.5	31.0
STOCK5S-18704	1.6	25	26.0
STOCK5S-38263	3.2	6.25	12.4
STOCK5S-28510	1.1	NA	44.4
BRZ127:5:E7	2.2	6.25	17.5
CAK201:43:E1	1.0	NA	>50
T5473027	1.5	6.25	8.8
T5817819	2.2	6.25	15.3
T5314847	4.8	12.5	19.4
T0520-7534	1.3	25	>50
T5990785	1.0	NA	42.9
T0509-0656	1.1	NA	>50
T5407434	1.4	6.25	12.5
T5845297	3.2	6.25	12.3
T0505-5074	3.4	12.5	10.2
T5227647	5.4	6.25	6.5
T5263000	3.7	6.25	8.4
T5544185	1.3	25	39.2
T5410513	1.1	NA	42.5
T5547254	1.1	NA	53.7
T5548412	1.4	25	46.6
T5621982	3.9	3.125	4.5
T5703243	1.1	NA	>50
T5801187	1.2	25	47.8
T5848463	1.6	25	46.1
T5929635	1.7	25	25.7
T6048356	1.4	25	26.4
T6092106	1.2	25	43.4
T6224950	1.0	NA	>50
T6225499	1.0	NA	>50
T6240036	2.4	12.5	23.7
T0505-3851	2.8	25	38.8
T0515-2003	4.3	25	22.6
T5226301	1.0	NA	22.1
T5383989	1.2	25	>50
T5383642	1.2	12.5	26.2
T5540719	1.3	25	30.2
T5541143	1.0	NA	26.1
T5985287	1.3	12.5	23.4
T6066027	1.5	12.5	27.1
T5650357	3.0	25	34.3
T5700438	3.1	6.25	8.3
T5731482	3.0	25	42.0
T5734439	1.6	25	22.4

T5872380	3.0	25	26.4
T5872712	1.6	25	42.5
T5872760	1.5	12.5	30.9
T6004974	1.2	25	37.0
T6117781	3.7	25	31.8
T6214849	4.9	12.5	15.3
T6248417	1.2	25	41.6
T6238737	1.2	25	39.7
5144511	1.0	NA	>5
5421628	1.0	NA	>5
5546507	1.0	NA	>5
5571547	1.0	NA	>5
5574341	1.0	NA	>5
5618307	1.0	NA	>5
6049586	1.0	NA	>5
6155812	1.0	NA	>5
7821616	1.0	NA	>5
7978712	0.9	NA	>5
CGX-02167237	1.0	NA	>5
CGX-03309303	1.0	NA	>5
033_0221_0172	1.0	NA	>5
11G-031	1.0	NA	>5
STOCK3S-28947	1.0	NA	2.7
STOCK3S-00823	1.1	NA	>5
STOCK1S-65123	1.0	NA	>5
STOCK1S-64351	1.1	NA	>5
STOCK3S-11937	2.9	2.5	2.1
STOCK3S-76410	2.3	2.5	3.0
STOCK1S-93075	1.1	NA	>5
STOCK1S-54857	0.9	2.5	>5
STOCK4S-38774	1.0	NA	>5
STOCK4S-62985	1.4	2.5	4.0
STOCK6S-15817	4.4	1.25	1.5
STOCK4S-60288	1.0	NA	3.2
STOCK5S-42531	3.3	0.625	1.2
STOCK5S-82907	4.3	1.25	1.6
STOCK5S-69744	1.0	NA	>5
STOCK5S-85745	1.0	NA	>5
STOCK6S-01108	1.0	NA	>5
STOCK6S-03252	4.2	1.25	>5
STOCK6S-41308	1.0	NA	>5
STOCK6S-32250	2.8	0.313	0.3
STOCK6S-37013	4.1	1.25	1.6
STOCK6S-42437	4.3	1.25	1.5
STOCK6S-38758	2.9	0.313	0.6
STOCK6S-35180	3.4	0.625	1.6
STOCK6S-41114	1.1	NA	>5
AF-399/09020026	0.9	NA	>5
STOCK1S-31919	4.2	0.313	0.8
STOCK1S-42137	5.7	0.156	0.2
STOCK1S-13735	12.4	12.5	13.6
STOCK1S-04771	7.0	12.5	11.6
STOCK3S-56977	0.9	NA	18.1
STOCK3S-92457	2.3	6.25	13.0
STOCK3S-87227	1.4	12.5	17.9
STOCK4S-04391	8.5	12.5	>25
STOCK1S-13266	1.5	12.5	>25
5464668	3.9	5	5.0

5558382	5.2	1.25	1.3
6374290	2.0	5	5.7
STOCK4S-44678	0.6	NA	2.3
STOCK6S-03600	1.5	10	8.8
STOCK3S-38373	1.5	5	7.3
STOCK4S-34434	2.3	0.625	5.7
T5496829	1.5	2.5	2.3

Table S2. Viability of *T. b. gambiense* STIB930, *T. b. brucei* GUSone and *T. b. rhodesiense* STIB900 after 40 hours drug exposure. The initial parasite density was 3.5×10^5/ml in the Alamar blue assay.

Compound	STIB930 IC_{50} [µg/ml]	STIB900 IC_{50} [µg/ml]	GUSone IC_{50} [µg/ml]
DIP-01	25.5	26.9	27.1
DIP-02	0.42	0.66	0.97
DIP-03	0.04	0.11	0.15
DIP-04	28.3	37.6	36.1
DIP-05	3.1	2.9	8.9
DIP-06	0.24	0.56	2.94
DIP-07	0.25	0.40	0.95
DIP-08	2.7	2.3	7.5
DIP-09	3.6	8.3	19.2
DIP-10	0.89	0.96	2.11
DIP-11	2.0	1.1	5.4
DIP-12	3.6	4.1	10.0
DIP-13	24.5	4.6	5.4
DIP-14	9.9	8.7	26.2
DIP-15	3.6	4.6	9.2
DIP-16	1.7	3.8	13.1
DIP-17	3.3	3.9	8.2
DIP-18	4.8	3.5	7.1
DIP-19	0.22	0.24	0.61
DIP-20	2.4	3.2	8.5
DIP-21	0.83	1.37	3.98
DIP-22	1.1	1.5	3.9
DIP-23	2.1	2.9	9.3
DIP-24	1.4	1.2	2.3
DIP-25	3.0	2.6	4.9
DIP-26	1.7	2.0	3.6
DIP-27	2.4	4.3	6.7
DIP-28	1.7	2.2	4.7
Melarsoprol	0.006	0.008	0.009
Pentamidine	0.003	0.005	0.006

Table S3. VSG loss triggered by *cis*-aconitate (CA). Dose response of CA (5 – 20 mM). Triplicate samples were analysed by flow cytometry. The median fluorescence intensity (MFI) was calculated for all datasets and is given in the table together with the corresponding means and standard deviations. The ratio of the mean of CA treated cells to untreated cells is given as T/B. StDev: standard deviation.

		18 hours						24 hours					
	Conc. mM	Triplicates of MFI			Mean	StDev	T/B	Triplicates of MFI			Mean	StDev	T/B
untreated		61.0	61.4	56.9	59.8	2.5	B	38.0	47.2	48.3	44.5	5.7	B
CA	5	156	144	138	146.0	9.2	2.4	137	143	128	136.0	7.5	3.1
	10	228	233	235	232.0	3.6	3.9	310	355	306	323.7	27.2	7.3
	20	311	341	313	321.7	16.8	5.4	441	472	396	436.3	38.2	9.8

Table S4. VSG loss for DIP compounds. For all compounds and concentrations indicated triplicate samples were analysed by flow cytometry. The median fluorescence intensity (MFI) was calculated for all datasets and is given in the table together with the corresponding means and standard deviations. The ratio of the mean of treated cells to untreated cells is given as T/B. *CA was tested at 20 mM. StDev: standard deviation.

		18 hours						24 hours					
	Conc. µg/ml	Triplicates of MFI			Mean	StDev	T/B	Triplicates of MFI			Mean	StDev	T/B
untreated		77.4	93.3	88.4	86.4	8.1	B	18.7	33.8	78.4	43.6	31.0	B
CA	20*	357	259	431	349.0	86.3	4.0	598	698	779	691.7	90.7	15.9
DIP-01	5	135	165	229	176.3	48.0	2.0	131	173	189	164.3	30.0	3.8
	10	196	235	247	226.0	26.7	2.6	161	259	242	220.7	52.4	5.1
	20	393	393	415	400.3	12.7	4.6	469	482	495	482.0	13.0	11.1
DIP-02	0.156	124	116	93.5	111.2	15.8	1.3	121	111	77.9	103.3	22.6	2.4
	0.323	150	120	112	127.3	20.0	1.5	118	124	99.1	113.7	13.0	2.6
	0.625	384	393	391	389.3	4.7	4.5	359	523	482	454.7	85.3	10.4
DIP-03	0.039	71.2	83.9	85.2	80.1	7.7	0.9	20.3	40.3	74.3	45.0	27.3	1.0
	0.078	120	108	94.1	107.4	13.0	1.2	24.5	47.5	69.9	47.3	22.7	1.1
	0.156	477	528	485	496.7	27.4	5.7	292	352	343	329.0	32.4	7.5
DIP-07	0.625	104	114	119	112.3	7.6	1.3	65.1	27.7	24.7	39.2	22.5	0.9
	1.25	172	138	145	151.7	18.0	1.8	116	75.3	39.4	76.9	38.3	1.8
	2.5	615	614	649	626.0	19.9	7.2	588	530	552	556.7	29.3	12.8
DIP-19	0.323	112	76.9	91.2	93.4	17.7	1.1	147	139	135	140.3	6.1	3.2
	0.625	114	116	68.8	99.6	26.7	1.2	161	166	151	159.3	7.6	3.7
	1.25	200	185	232	205.7	24.0	2.4	390	254	267	303.7	75.0	7.0

Table S5. VSG loss of standard drugs. For all compounds and concentrations indicated triplicate samples were analysed by flow cytometry. The median fluorescence intensity (MFI) was calculated for all datasets and is given in the table together with the corresponding means and standard deviations. The ratio of the mean of treated cells to untreated cells is given as T/B. *CA was tested at 20 mM. StDev: standard deviation.

		24 hours					
	Conc. ng/ml	Triplicates of MFI			Mean	StDev	T/B
untreated		13.9	17.8	16.5	16.1	2.0	B
CA	20*	109	170	106	128.3	36.1	8.0
Pentamidine	1.25	16.7	15.5	14.4	15.5	1.2	1.0
	2.5	18.9	19.1	22.1	20.0	1.8	1.2
	5	73.7	70.4	63.9	69.3	5.0	4.3
Melarsoprol	5	7.14	10.1	8.88	8.7	1.5	0.5
	10	26.9	25.3	16.9	23.0	5.4	1.4
	20	168	174	149	163.7	13.1	10.2
Nifurtimox	2,500	11.9	11.9	10.9	11.6	0.6	0.7
	5,000	49.5	41.9	40.6	44.0	4.8	2.7
	10,000	225	245	222	230.7	12.5	14.3

Table S6. Primers used in quantitative reverse transcription PCR.

Primer name	Sequence	Gene(s) amplified
**C1_fwd_qPCR**	ttgtgacgacgagagcaaac	Tb927.10.12970
**C1_rev_qPCR**	agtggttgaacgccaaatgc	ditto
**Proc_f_qPCR**	tggcacctcgttccctttatc	Tb927.6.450, Tb927.6.480, Tb927.6.510, Tb927.6.520, Tb927.10.10250, Tb927.10.10260
**Proc_r_qPCR**	aaatcccacgccagagaagag	ditto

Supplementary information figures

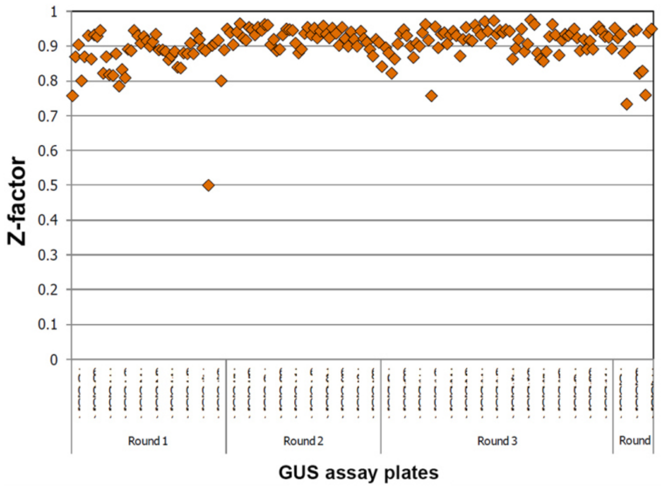

Figure S1. Z'-factor of each plate in the MTS. The high Z'-factor of 0.9 in average indicates a highly robust assay. Controls: trypanosomes with *cis*-aconitate (CA) served as the positive control and trypanosomes without any drug served as the negative control.

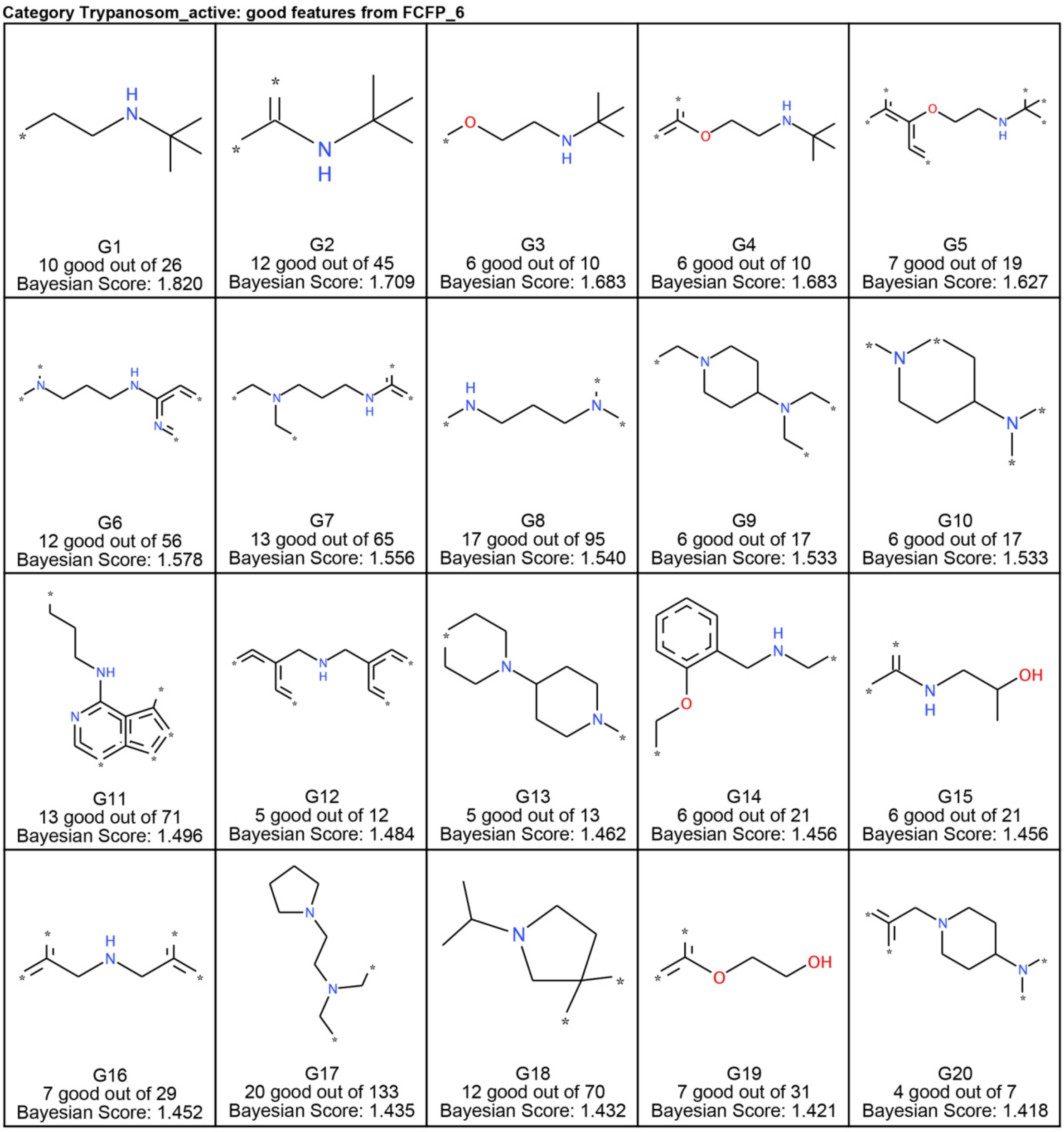

Figure S2. Substructure features significantly enriched in compounds activating GUS compared to the total test set of 7495 compounds. The 212 active compounds and all other compounds screened were designated as active and inactive, respectively. Default descriptors including FCFP_6 fingerprints were used as molecular descriptors to identify features enriched in the active compounds, using the Pipeline Pilot implementation of a two-class Bayesian categorization model (Biovia, San Diego, USA).

Figure S3: Anti-trypanosomal control drugs tested in the GUS reporter assay and the viability assay. The standard drugs melarsoprol, pentamidine, suramin, nifurtimox and fexinidazole were tested for viability (Alamar Blue) and for GUS activation with a 40 hour drug exposure time and initial parasite density of 5×10^5 GUSone trypanosomes /ml. All compounds were tested over a ≥1,000-fold concentration range with 2x dilution steps. The dose response plots are from a single experiment. Among the standard trypanocidal drugs tested, only melarsoprol gave a GUS signal (S/B>2). No GUS activation was observed following exposure to pentamidine, suramin, nifurtimox and fexinidazole, including non-toxic concentrations. At highly trypanocidal concentrations the GUS signal is below the background signal (trypanosomes without drug) resulting in S/B ratio <1.

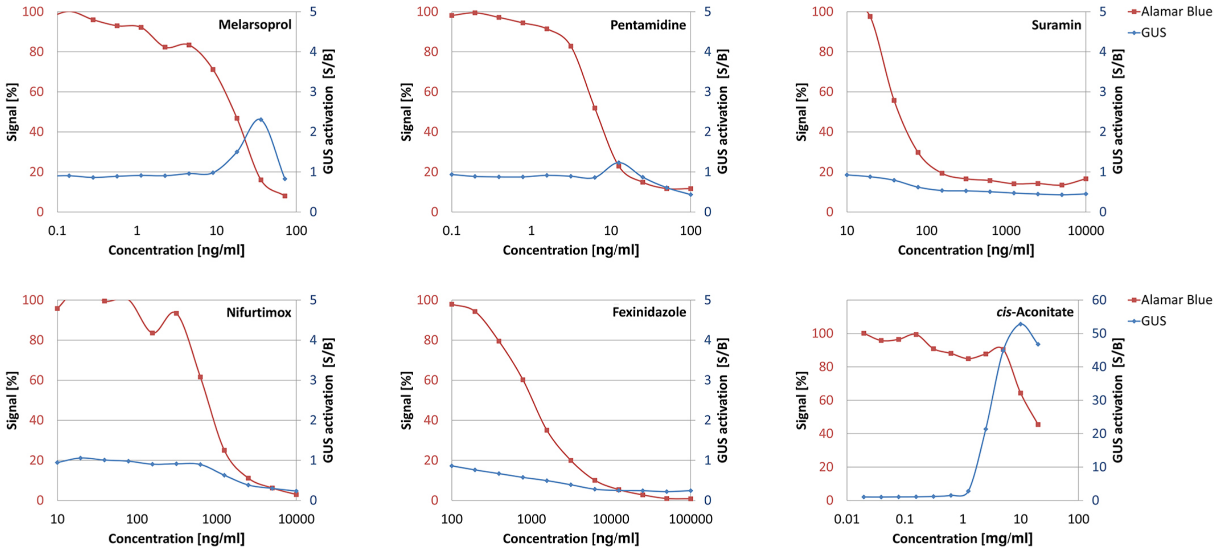

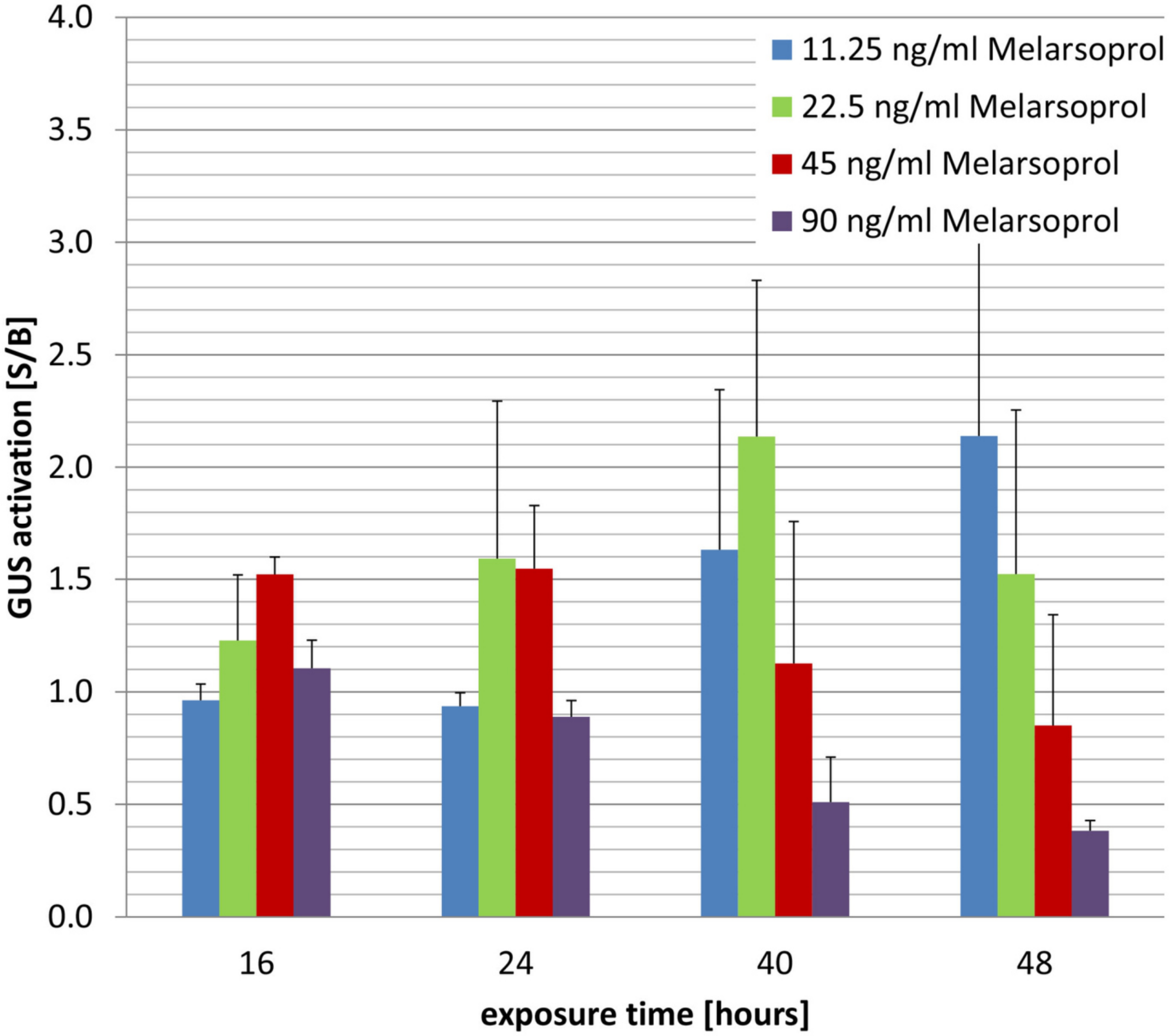

Figure S4. Melarsoprol induces GUS expression up to 2x over background. The starting trypanosome density was 8×10^5 parasites/ml for 16 h and 24 h, 5×10^5 parasites/ml for 40 h and 2.5×10^5 parasites/ml for 48 h exposure. The S/B was 2 fold at 22.5 ng/ml after 40 h exposure or at 11.25 ng/ml after 48 h. S/B: Ratio of GUS signal of melarsoprol-treated trypanosomes to GUS signal of untreated trypanosomes. Bars indicate standard deviations of S/B from at least three independent assays.

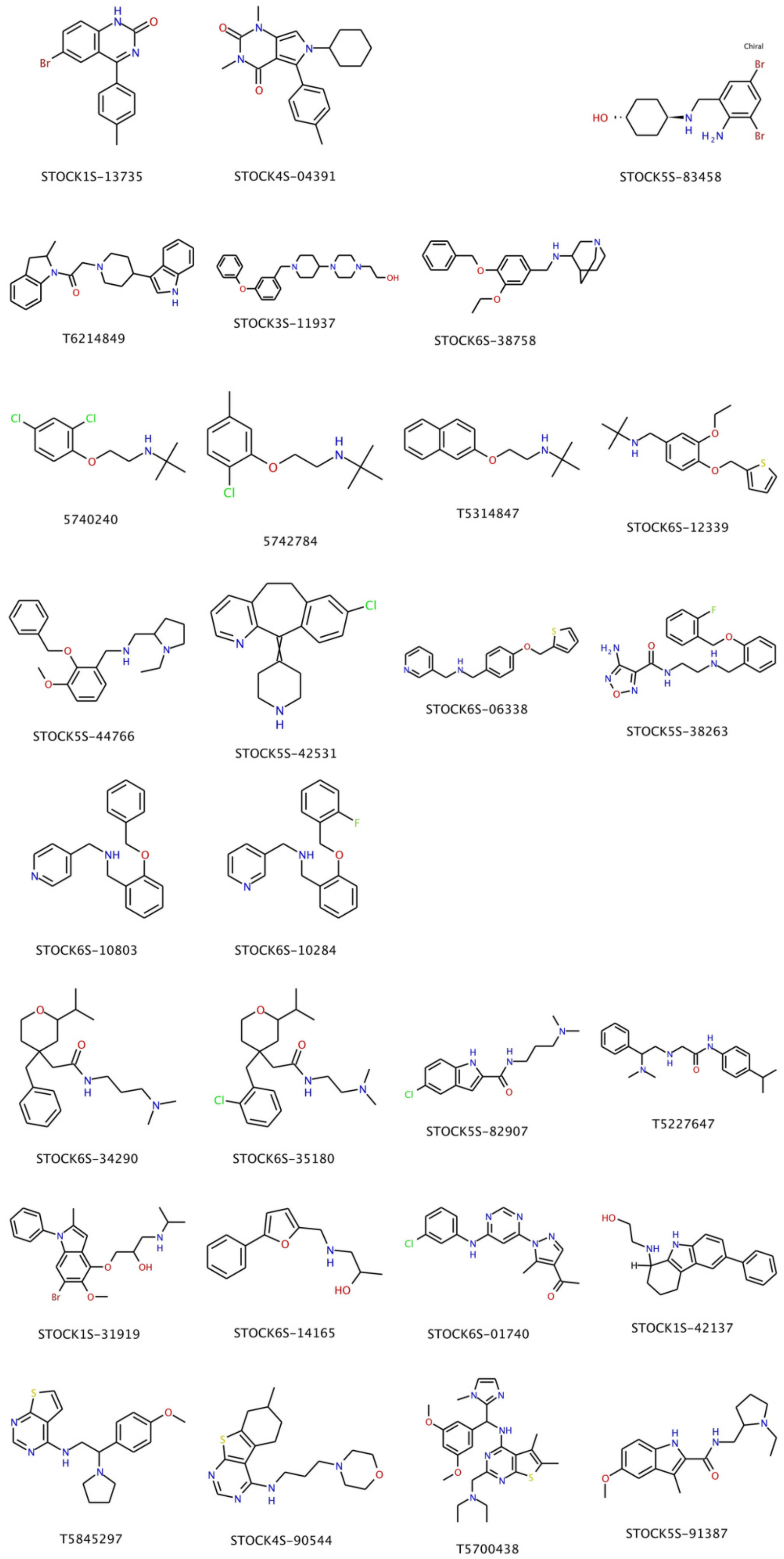

Figure S5. Chemical structures of 28 DIP-compounds with PubChem ID. Structures were grouped by similar features or certain sub-sets.

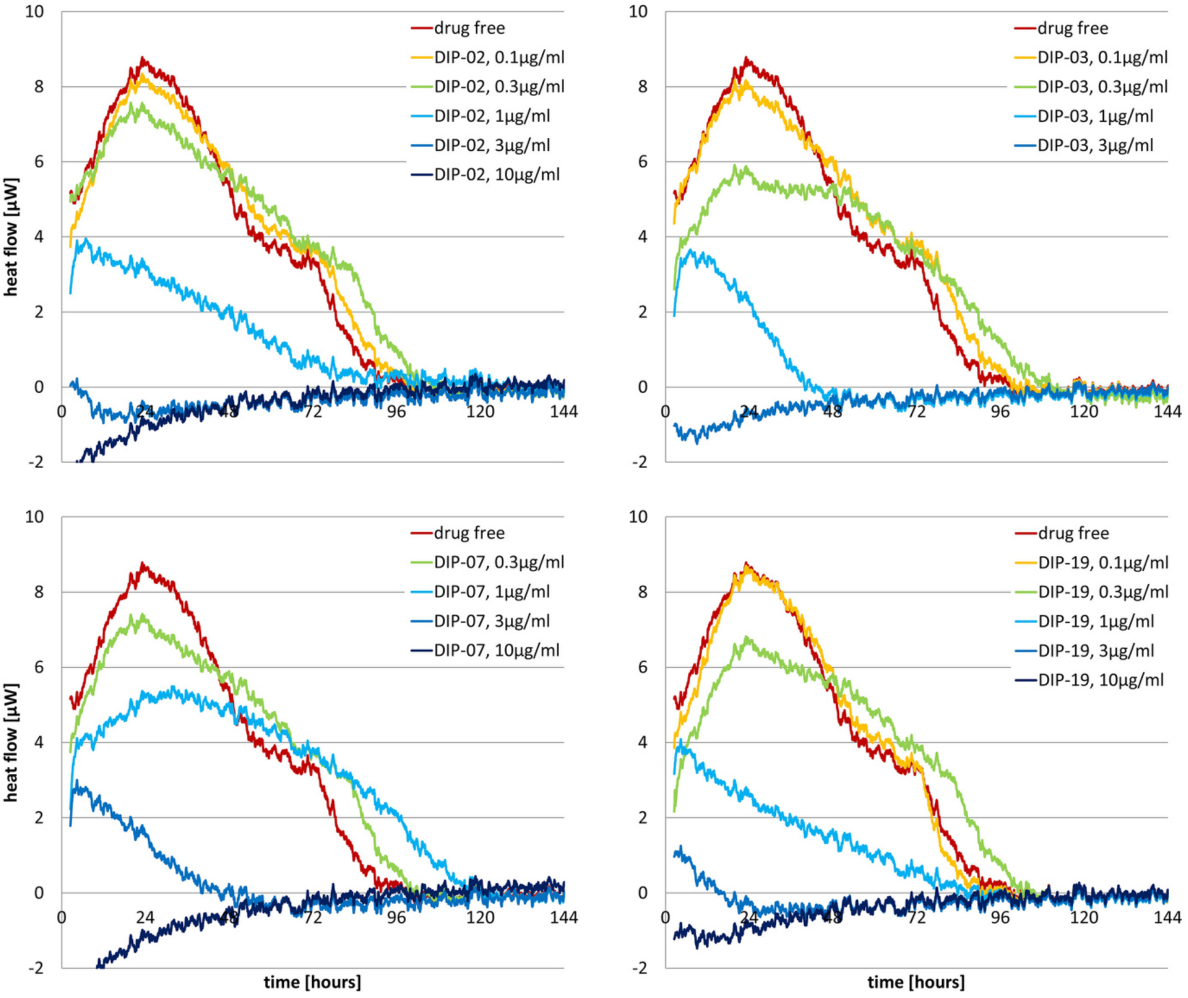

Figure S6. Microcalorimetric analysis of 4 DIP compounds at various concentrations. Details of the assay are provided in the methods section. The time to kill was short: at the highest concentration the DIP compounds killed over 99% of the cells before the equilibration time (i.e. ≤4 h). At the second highest concentration the parasites were killed within 48 h. DIP-01 is insoluble at the high concentrations required for fast killing and was therefore omitted from the experiment.

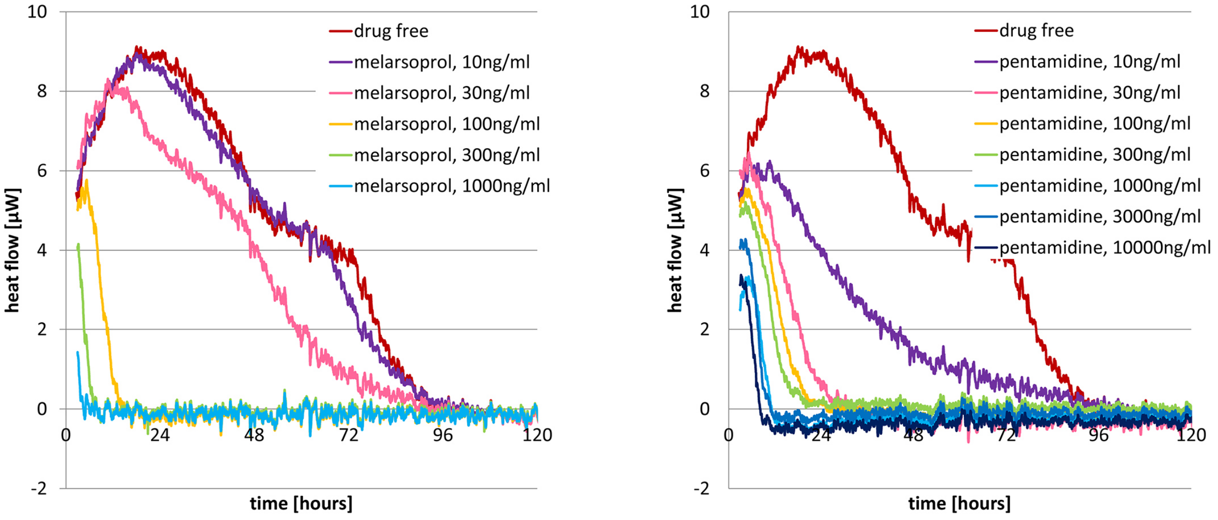

Figure S7. Microcalorimetric analysis of trypanocidal control compounds at various concentrations. Dose dependency and time of drug action are different for melarsoprol and pentamidine. Pentamidine is slower acting at high drug concentrations (>10-fold IC_{50}) and a wider concentration range is required to cover low to strong inhibition than for melarsoprol and the 4 DIP compounds (Figure S6).

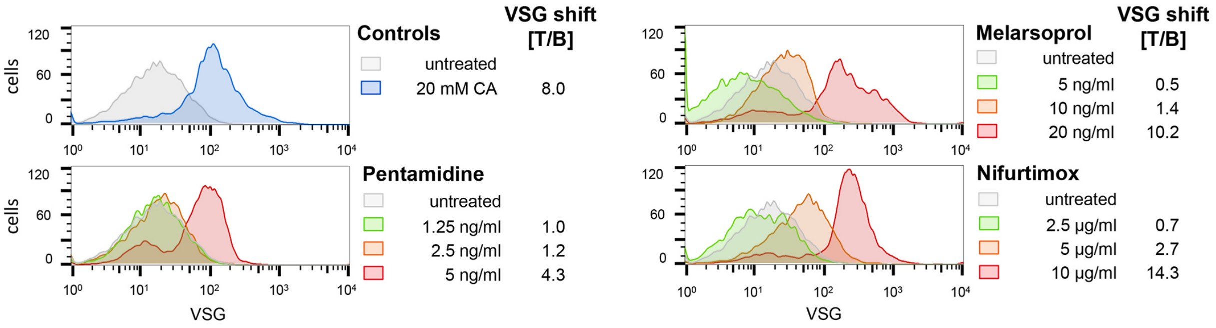

Figure S8. Loss of VSG of standard drugs at 37°C. The density of the VSG coat was monitored by staining with anti-VSG221 antibodies and flow cytometry. Assays were performed in triplicate with a 24 h drug exposure time. To display the dose-dependent VSG shifts, representative curves of different drug concentrations were plotted together with the corresponding untreated control. MFI: median fluorescence intensity. T/B: VSG changes are expressed as a ratio of the MFI after treatment to the MFI of the untreated control. Full flow cytometry data are available in Table S5.
